# Human TGFalpha-derived peptide TGFalphaL3 fused with superantigen for immunotherapy of EGFR-expressing tumours

**DOI:** 10.1186/1472-6750-10-91

**Published:** 2010-12-22

**Authors:** Quanbin Xu, Xiaojuan Zhang, Junjie Yue, Chuanxuan Liu, Cheng Cao, Hui Zhong, Qingjun Ma

**Affiliations:** 1 Beijing Institute of Biotechnology, Taiping Road 27, Beijing, PR China; 2 Institute of Genetics and Developmental Biology, No.1 West Beichen Road, Chinese Academy of Sciences, Beijing, PR China

## Abstract

**Background:**

Monoclonal antibodies have been employed as targeting molecules of superantigen for the preclinical treatment of a variety of tumours. However, other targeting molecules, such as tumour-related ligands or peptides, are less exploited. Here, we tested other targeting molecules by genetically fusing the third loop of transforming growth factor alpha (TGFalphaL3) to mutant staphylococcal enterotoxin A (SEA_D227A_).

**Results:**

The resultant fusion proteins were expressed in *E. coli *and purified to homogeneity through a Ni-NTA affinity column. Fusion protein TGFalphaL3SEA_D227A _can promote splenocyte proliferation to a level comparable to recombinant SEA (rSEA) and bind to EGFR-expressing tumour cells in an EGFR-dependent way. Consistent with these observations, TGFalphaL3SEA_D227A _exerted an inhibitory effect on the growth of EGFR-expressing tumour cells both *in vitro *and *in vivo*. Notably, significant infiltrations of CD8^+ ^and CD4^+ ^T cells were detected in the tumour tissues of these C57BL/6 mice treated with TGFalphaL3SEA_D227A, _suggesting the involvement of T cells in this tumour-inhibitory process.

**Conclusions:**

The data here showed that TGFαL3 is capable of targeting superantigen to tumours and exerting an inhibitory effect on tumour growth, which enables TGFαL3SEA_D227A _to be an attractive candidate for the immunotherapy of EGFR-expressing tumours.

## Background

Superantigens (SAgs) are microbial proteins with the capacity to activate a large fraction of T cells [1]. The cellular receptors for SAgs are major histocompatibility complex (MHC) class II molecules and T-cell antigen receptors (TCR) [2-4]. SAgs can bind to the TCR β subunit and activate T cells independently of their CD4 or CD8 phenotype when presented by MHC class II molecules [5,6]. Activated T cells secrete a variety of cytokines, such as TNFα, INFγ, IL-1, IL-2, IL-6, IL-8 and IL-12 [7,8]. Staphylococcal enterotoxin type A (SEA) is a protein exotoxin secreted by certain strains of Staphylococcus aureus, which was demonstrated to direct cytotoxic T cells (CTLs) against MHC class II expressing tumour cells effectively [9]. However, MHC class II positive tumours only represent a minor fraction of the most frequent human tumours. To introduce a novel binding specificity in SEA, a monoclonal antibody (mAb) specific for colon carcinoma antigen C215 was initially conjugated to SEA, and the resultant conjugate Fab-SEA could lyse antigen expressing tumour cells significantly *in vitro *[10]. To date, SEA fused to various mAb have been subjected to preclinical treatment of many tumour types, some of which have finished phase I or phase II clinical trials, such as C242Fab-SEA (PNU-214565) and 5T4FabV13SEAD227A (ABR-214936)[11-14].

EGFRs are over-expressed in a variety of human tumour cells, including breast, head, neck, gastric, colorectal, oesophageal, prostate, bladder, renal, pancreatic, ovarian and nonsmall cell lung cancer (NSCLC) [15]. Moreover, the degree of EGFR over-expression is associated with an advanced tumour stage and resistance to standard therapies [16-19]. EGFR-targeted therapies have been proven to be successful by using monoclonal antibodies (i.e. Herceptin) or tyrosine kinase inhibitors (i.e. gefitinib). Unfortunately, not all patients bearing tumours with over-expression of EGFR or Her2 respond to those drugs. Only about 10% of NSCLC patients responded clinically to gefitinib; somatic mutations within the EGFR kinase domain were exclusively observed in lung cancer cells in these patients [20,21].

Human transforming growth factor alpha (hTGFα) is a native ligand co-overexpressed with its receptor EGFR in many human tumours [15]. hTGFα consists of three loops, the third of which (TGFαL3) retains binding ability to EGFR but lacks mitogenic activity [22]. Binding of TGFαL3 to EGFR is not affected by mutations in the EGFR kinase domain, which suggests a function for TGFαL3 as a targeting molecule, where ligand/receptor induced internalisation is not required. Moreover, compared to mAbs, TGFαL3 is presumably less antigenic, thereby maintaining a longer circulating half-life. These properties enable TGFαL3 to be an attractive targeting molecule for the superantigens, which function only when presented on the cell surface. However, the binding ability of TGFαL3 to its receptor is relatively weaker than that of mAbs to antigen. This raises the question whether the affinity of a small peptide is strong enough to bring SAgs to tumours *in vivo*. Here, we tested this idea by fusing TGFαL3 to SEA (D227A), a mutant of SEA defective for MHC-II [23]. Encouragingly, we found that the resultant fusion protein TGFαL3SEA_D227A _could bind to EGFR-expressing tumour cells and exhibited an apparent growth inhibitory effect on the tumour cells, both *in vitro *and *in vivo*. T cells likely mediated the inhibitory effect, which was suggested by the significant infiltration of CD8^+ ^and CD4^+ ^T cells in fusion protein-treated tumour tissues.

## Results

### Construction and expression of fusion proteins

**Figure 1:**
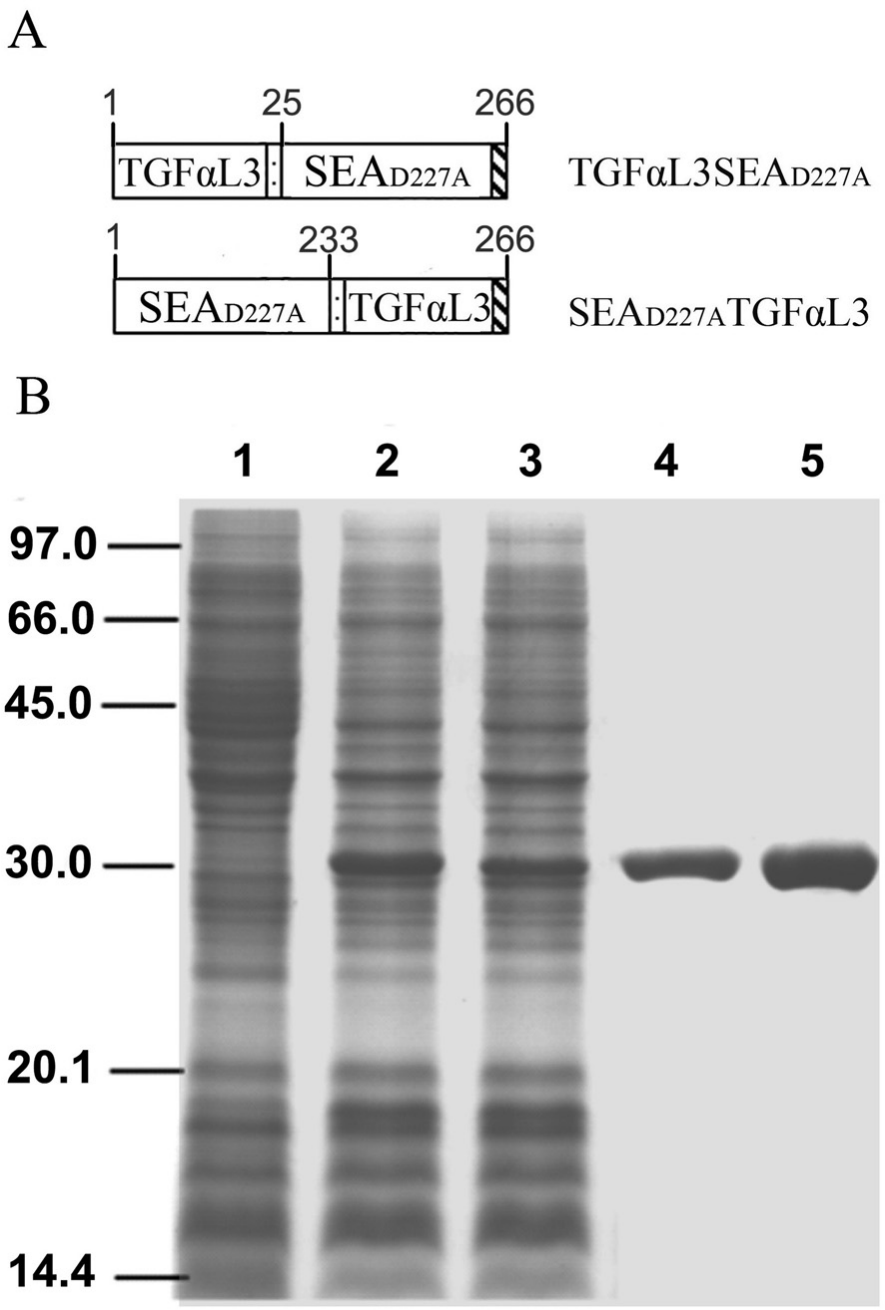
**Expression and purification of fusion proteins**. (A) Schematic presentation of fusion proteins. TGFαL3 was fused to the N -terminal or the C-terminal of SEAD_227A _with 8 an eight-amino acids linker. The linker sequence (GGSGSGGG) was indicated by a dotted rectangle. All fusion proteins were tagged with a six histidinesix-histidine tag at the C-terminal; TGFαL3, the third loop of TGFα; SEA_D227A_, site mutated SEA (D227A). (B) Expression and purification of fusion proteins. *E. coli *BL21 (DE3) cells were transformed with plasmids encoding fusion proteins, and then induced with 0.5 mM IPTG at 22°C overnight. The proteins were separated by a 15% SDS-PAGE gel and stained with Coomassie blue. Lane 1, total cell proteins (TCP) of BL21 (DE3) with pET-22b (+); lane 2, TCP of BL21 (DE3) with pET-22b-TGFαL3SEA_D227A_; lane 3, TCP of BL21 (DE3) with pET-22b-SEA_D227A_TGFαL3; lane 4, purified TGFαL3SEA_D227A_; lane 5, purified SEA_D227A_TGFαL3.

To test the effect of TGFαL3 fusion on SEA (D227A) activity, two sets of constructs, pET-22b-TGFαL3SEA_D227A _and pET-22b-SEA_D227A_TGFαL3, were made. These vectors are pET-22b (+)-derived bacterial expression vectors, which drive the expression of fusion protein TGFαL3SEA_D227A, _or SEA_D227A_TGFαL3, respectively (Figure 1A). Constructs were transformed into *E. coli *strain BL21 (DE3) and protein expression was induced by 0.5 mM IPTG. Proteins of interest with the expected molecular weight (30 kDa) were observed in total cell pellets (Figure 1B, lane 2, 3). About 55% soluble fusion proteins appeared in the spheroplast when bacteria cells were induced at 22°C (data not shown). Soluble fusion proteins were purified from the supernatant of sonicated bacterial pellets through a Ni-NTA affinity column. Purified proteins were further dialysed against PBS and concentrated by ultrafiltration. Coomassie blue staining after SDS-PAGE separation showed that both TGFαL3SEA_D227A _and SEA_D227A_TGFαL3 were purified to 95% homogeneity (Figure 1B, lane 4, 5).

### TGFαL3SEA_D227A _promotes splenocyte proliferation and binds to the EGFR

**Figure 2:**
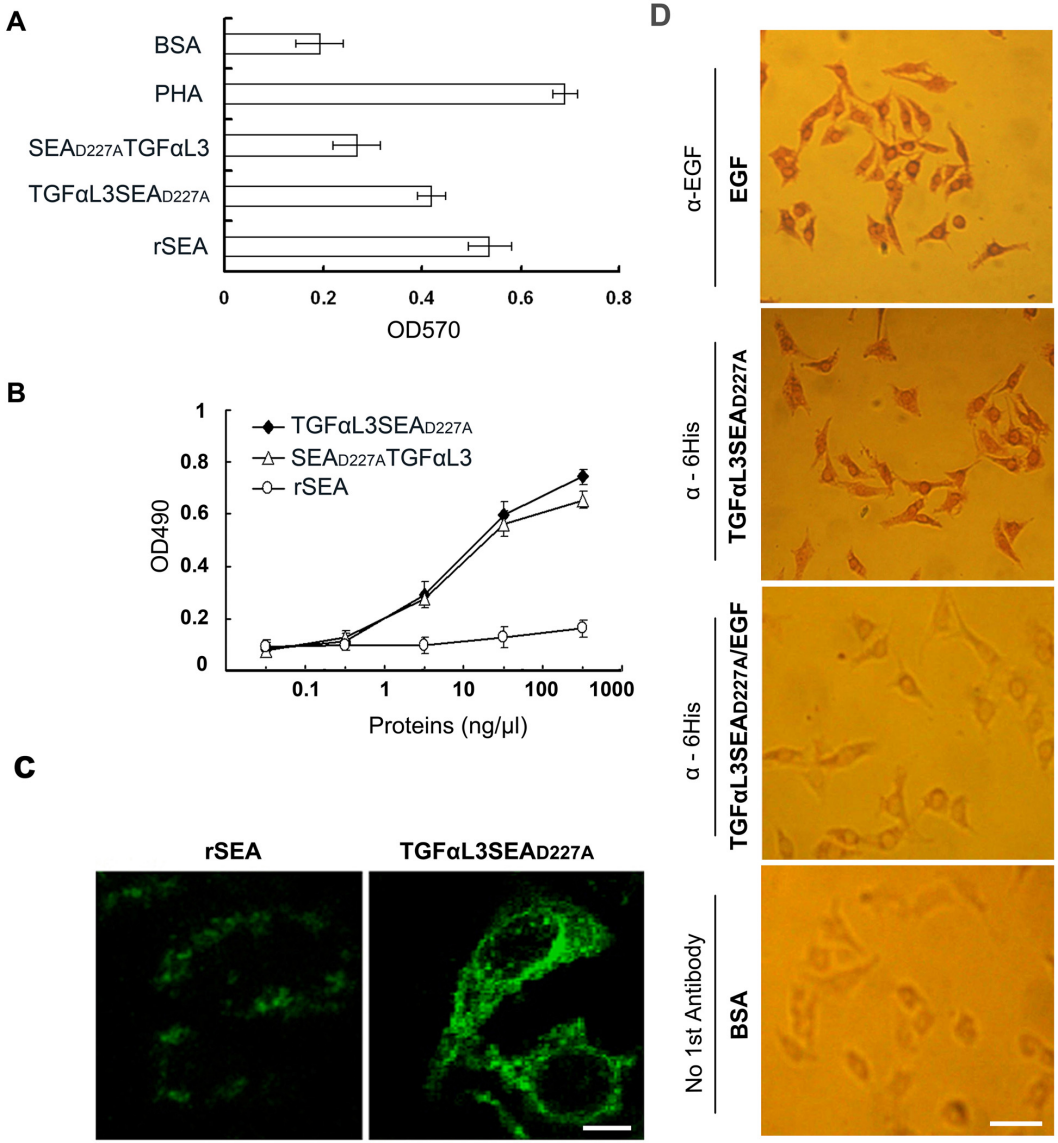
**TGFαL3SEAD227A promotes splenocyte proliferation and binds to the EGFR**. (A) Superantigenic activity of the fusion proteins. 1.0 × 10^5 ^freshly isolated splenocytes were seeded in each well in of a 96-well plate at a density of 1.0 × 10^5 ^cells/well. The cells were then incubated with 1.0 μg/ml TGFαL3SEA_D227A_, 1.0 μg/ml SEA_D227A_TGFαL3, 1.0 μg/ml rSEA, 25 μg/ml PHA or 1.0 μg/ml BSA at 37°C for 72 h, and then MTT solution was dispensed to each well and the optical density at 570 nm was measured. Each column shows the mean ± SD of triplicate samples. (B) The binding of fusion proteins to EGFR. A431 cells were cultured in a 96-well plate, and then fixed with 10% neutral formaldehyde at room temperature for 1 h. The bindings of cells to various concentrations of fusion proteins were detected by cell ELSIA (TGFαL3SEA_D227A _(black diamond), SEA_D227A_TGFαL3 (triangle), rSEA (circle). Each point shows the mean ± SD of triplicate samples. (C) Determining the association of proteins to the EGFR via immunofluorescence staining. A431 cells were seeded on a cover glass in a 12-well plate overnight, and then fixed with 10% neutral formaldehyde. The cells were treated with 50 ng/μl rSEA or TGFαL3SEA_D227A_, and subsequently incubated with anti-hexahistidine mAb and FITC-conjugated rabbit anti-mice IgG. Bar, 8 μm. (D) Binding specificity of TGFαL3SEA_D227A _to A431 cells. A431 cells were seeded and treated as (C). Then 50 ng/μl EGF, TGFαL3SEA_D227A, _a mixture of EGF (50 ng/μl) and TGFαL3SEA_D227A _(50 ng/μl), or PBS were applied to the A431 cells for two hours. After an extensive wash, cells were incubated using anti-EGF, anti-6His, or blank control in PBS (only secondary antibody applied) treated cells. The color was then developed using a standard protocol after the secondary antibody was applied. Bar, 50 μm.

To determine the proliferating effect of fusion proteins, mice splenocytes were incubated with TGFαL3SEA_D227A _or SEA_D227A_TGFαL3, rSEA, or PHA at 37°C for 72 hours. As showed in Figure 2A, TGFαL3SEA_D227A _maintained 77.8% of the proliferative activity of rSEA. As a control, rSEA and PHA induced strong proliferation of splenocytes. However, SEA_D227A_TGFαL3 showed only 49.8% of the proliferative activity of rSEA, implying that the TGFαL3 C-terminal fusion negatively affects SEA_D227A_'s activity.

To investigate whether fusion proteins efficiently bind to EGFR, A431 cells that were derived from a human epidermoid carcinoma characterised by high levels of EGFR expression, were incubated with different concentrations of 6His-tagged TGFαL3SEA_D227A _or SEA_D227A_TGFαL3, or rSEA (from 0.1 ng/μl to 1 ug/μl). The cells were then incubated with anti-6His tag antibody followed by HRP conjugated anti-mouse IgG. The results showed that both TGFαL3SEA_D227A _and SEA_D227A_TGFαL3 bind to A431 cells with a similar affinity. As a control, rSEA did not bind to A431 cells (Figure 2B). Similar results were also obtained through immunostaining of recombinant proteins via anti-6His antibody. TGFαL3SEA_D227A _predominantly localised to the cell surface, as seen by fluorescent staining (Figure 2C). Unexpectedly, there was some punctate staining in the cytoplasm, which seems inconsistent with the previous report that TGFαL3 is not mitogenic [22]. There are two possible explanations: first, the fusion protein could be imported into cells via an unknown mechanism; second, it is artificial staining caused by fixation or nonspecific binding.

Although both TGFαL3SEA_D227A _and SEA_D227A_TGFαL3 bind to EGFR more efficiently than rSEA, we still cannot rule out the possibility that the binding of the fusion protein to EGFR-expressing cells may not be mediated by EGFR but by other membrane proteins. To exclude this possibility, we conducted a ligand competition assay. As showed in Figure 2D, the binding of the fusion protein to A431 cells can be efficiently blocked by the addition of EGF, which binds exclusively to EGFR. This result strongly suggests that the binding of the fusion protein to A431 cells is mediated by its specific interaction with EGFR.

### TGFαL3SEA_D227A _inhibited tumour cell growth *in vitro*

**Figure 3:**
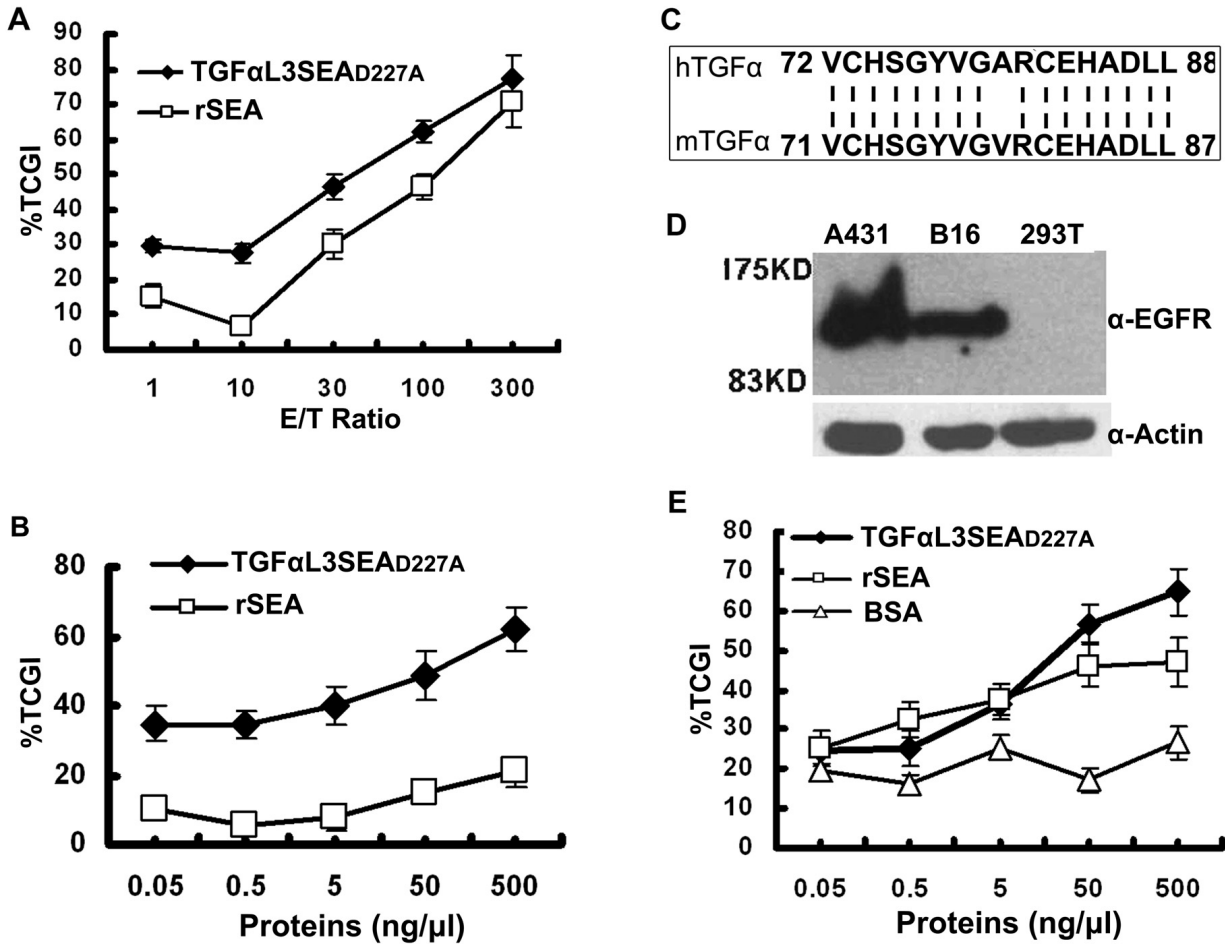
**TGFαL3SEA_D227A _inhibits tumour growth *in vitro***. (A) A431 cells were seeded into a 96-well plate at a density of 2.5 × 10^4 ^cells/well. Following an overnight incubation period, various ratios of effector cells and target cells were co-cultured with A431 cells in the presence of 5 ng/μl rSEA(squire) or TGFαL3SEA_D227A_(black diamond). Viable tumour cells were determined using an MTT assay, and the data were given as a percentage of tumour cell growth inhibition (TCGI). (B) A431 cells were plated in a 96-well plate at a density of 2.5 × 10^4 ^cells/well, followed by treatment with the indicated concentrations of TGFαL3SEA_D227A _(black diamond) and rSEA (square) at an E/T ratio of 30:1 *in vitro*. Viable tumour cells were determined as described above (A). (C) This figure indicates the alignment of the third loop of human TGFα and mice TGFα. (D)The protein level of EGFR in A431, B16 and HEK293T cells was detected by western blotting. (E) B16 cells, plated in a 96-well plate at a density of 2.5 × 10^4^, were treated identically to the A431 cells with the indicated dose of TGFαL3SEA_D227A _(black diamond), rSEA(square), and BSA (triangle).

Because TGFαL3SEA_D227A _has stronger proliferating activity than SEAD227ATGFαL3, we focused our interest on the activity of TGFαL3SEA_D227A_. To test whether TGFαL3SEA_D227A _bridges tumour cells and immune effecter cells together, which then leads to the lysis of tumour cells, A431 cells were initially co-cultured with increasing numbers of activated spleen cells in the presence of TGFαL3SEA_D227A_. As shown in Figure 3A, half inhibition of tumour cell growth by TGFαL3SEA_D227A _was achieved when the effector/target (E/T) ratio was 30:1. Under this E/T ratio, we found that TGFαL3SEA_D227A _inhibits A431 cell growth in a dose-dependent manner (Figure 3B). The third loop of human TGFα and mouse TGFα are highly conserved (94% of homogeneity) (Figure 3C). Further, murine melanoma B16 cells express comparable EGFR to that found in A431 cells (Figure 3D). This evidence suggests that TGFαL3SEA_D227A _also affects EGFR-expressing mouse tumours. Consistent with this hypothesis; the growth of B16 cells was remarkably restrained when co-incubated with TGFαL3SEA_D227A _(Figure 3E).

### TGFαL3SEA_D227A _inhibited tumour cell growth *in vivo*

**Figure 4:**
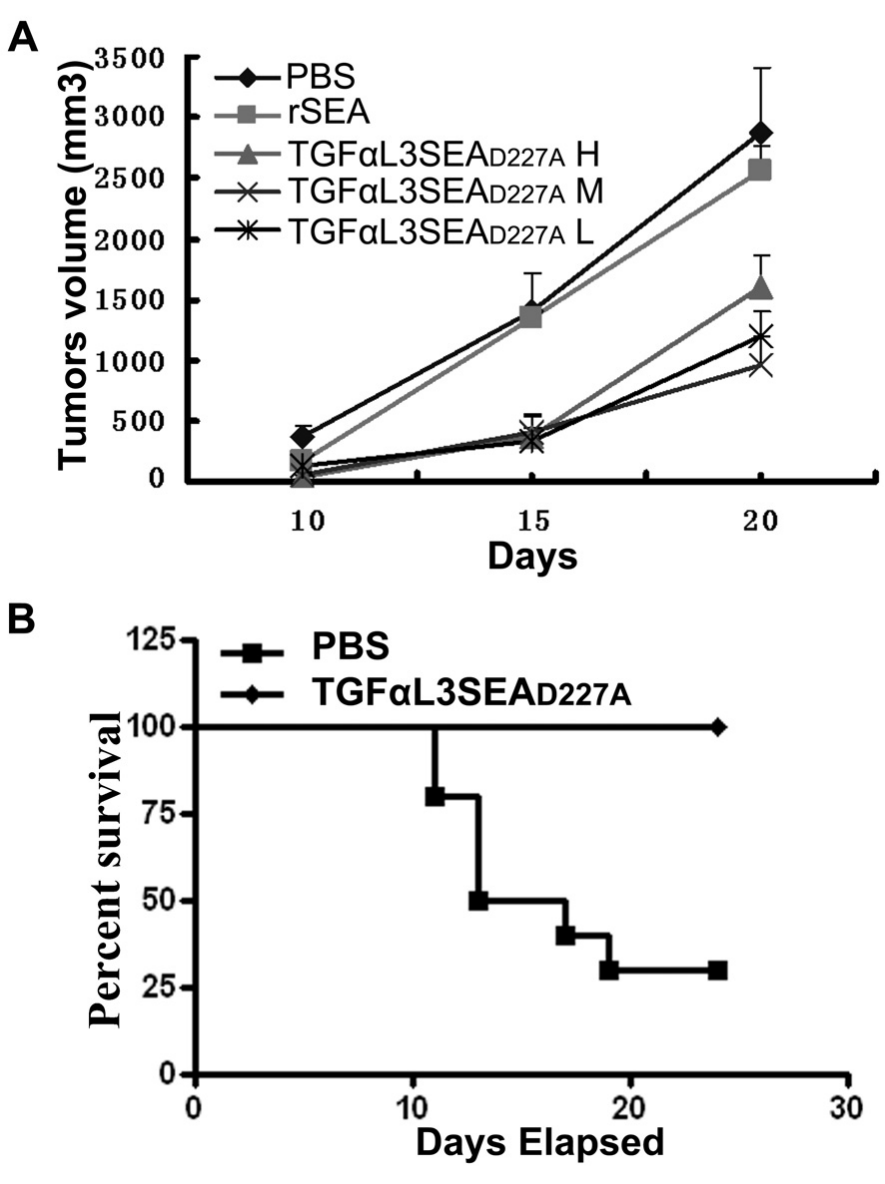
**TGFαL3SEA_D227A _inhibits tumour growth *in vivo***. (A) Thirty C57BL/6 mice were inoculated subcutaneously with 1.0 × 10^6 ^B16 melanoma cells. When the tumour length exceeded 0.5 cm, the mice were separated into equal-size groups and injected intraperitoneally with 6 pmol (asterisk), 60 pmol (multiplication), 600 pmol TGFαL3SEA_D227A _(black triangle), 60 pmol rSEA(black square) or PBS (black diamond), respectively, four times at one day intervals. The tumour sizes were measured with callipers at the time points indicated. The tumour volumes were calculated as (length × width^2^)/2. Each point shows the mean ± SD of the tumour volume from ten treated mice. (B) Twenty mice were inoculated with 1.0 × 10^6 ^B16 melanoma cells each and then randomised into two groups. The mice were treated with 60 pmol TGFαL3SEA_D227A _(black diamond) or PBS (black square) according to the protocol above when the tumour length exceeded 1 cm. The survival percentages were analysed by Kaplan Meier Plot.

To test whether the peptide TGFαL3 can bring SAgs to tumours *in vivo*, we evaluated the anti-tumour effect of TGFαL3SEA_D227A _in mouse models. C57BL/6 mice were subcutaneously inoculated with B16 cells. When the length of tumours exceeded 5 mm, TGFαL3SEA_D227A _was administrated intraperitoneally four times at one-day intervals. Tumour sizes from each mouse were recorded at the designed intervals. Consistent with the *in vitro *results, we found that all doses of TGFαL3SEA_D227A _effectively retarded tumour growth. The maximum tumour growth inhibition percentage was 84.1%, which was observed in 60 pmol of TGFαL3SEA_D227A _treated group at the 15th day (Figure 4A). As a control, rSEA only exerted a marginal effect on tumour growth throughout the therapeutic process (Figure 4A).

To investigate the effect of TGFαL3SEA_D227A _on mouse survival, we treated mice with 60 pmol of fusion drug identically when the tumours' length exceeded 1 cm. The survival of mice was checked daily, and the percent survival of the two groups were analysed by Kaplan Meier Plot. Throughout the experiment, no deaths were observed in the TGFαL3SEA_D227A _group, while only 30% of the mice in the PBS control group survived (Figure 4B). This data showed that TGFαL3SEA_D227A _treatment could significantly increase the survival time for tumour-bearing mice.

### TGFαL3SEA_D227A _induced immunoresponse against tumour cells

**Figure 5:**
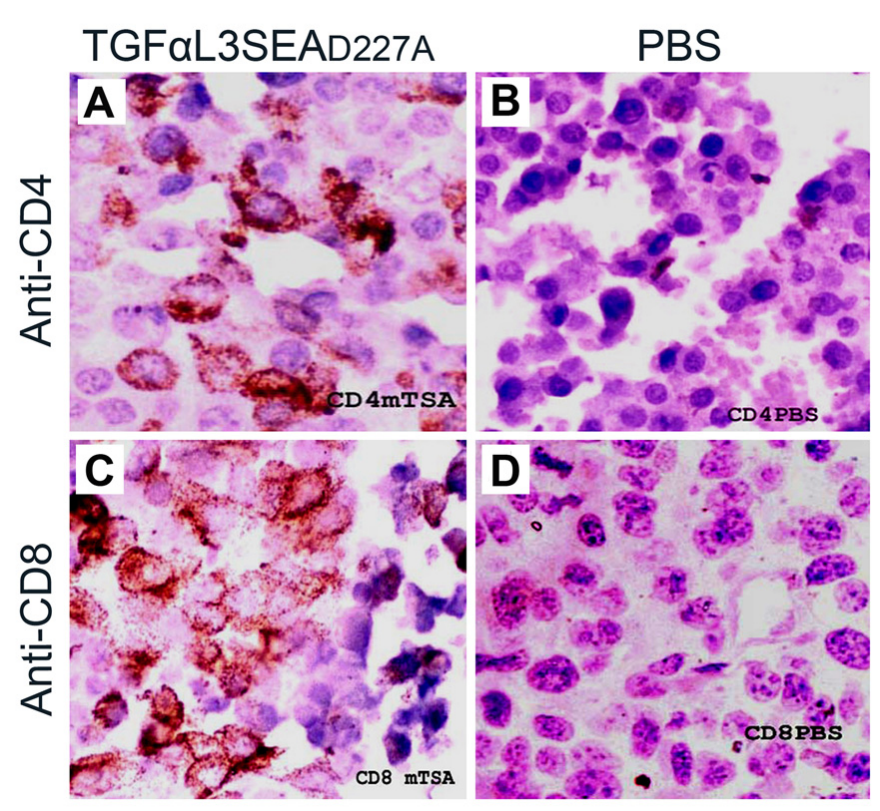
**TGFαL3SEA_D227A _recruits T cells to the tumour tissue**. Tumour tissues were excised from mice treated with 60 pmol TGFαL3SEA_D227A _or PBS after 24 days. The histochemical samples were prepared in a standard way, followed by staining with antibodies against the CD4 molecule (A and B) or the CD8 molecule (C and D). CD4^+ ^and CD8^+ ^T cells in the PBS control group were shown in figure 5B and 5 D, respectively; CD4^+ ^and CD8^+ ^T cells in the TGFαL3SEA_D227A _group are shown in figure 5A and 5C, respectively.

Recruitment of antigen-specific lymphocytes to tumour tissue is a major goal in tumour immunotherapy. Both CD4^+ ^and CD8^+ ^T cells were targeted by colon cancer specific mAb conjugated SEA, to lyse colon cancer *in vivo *[10]. To investigate whether TGFαL3SEA_D227A _inhibited tumour growth in a similar way, we examined for infiltration of CD4^+ ^and CD8^+ ^T cells in fusion protein-treated tumour tissue. Not surprisingly, both CD4^+ ^and CD8^+ ^T cells were observed around the tumour tissues (Figure 5AC), while recruitment of the same subtype of T cells was almost non-existent in the PBS control group (Figure 5BD). These results indicated that the tumour inhibitory effect induced by TGFαL3SEA_D227A _was, at least partially, attributed to the infiltration of both CD4^+ ^and CD8^+ ^T cells.

## Discussion

The application of SAgs for tumour therapy has been attempted for decades. The core aim of these applications is to specifically bring SAgs to tumour cells via various strategies, including tumour specific monoclonal antibody targeted or membrane anchored SAgs [10,24,25]. To date, mAb targeted superantigens have been successfully applied to several types of tumours, including B lymphocytic leukaemia, neuroblastoma, non-small cell lung cancer, melanoma, and renal cell carcinoma [13,26-29]. Two of them are on the way to clinical phase trials, including C242Fab-SEA (phase I) [14] and 5T4FabV13SEAD227A (phase II) [13]. Notably, human anti-mouse antibody (HAMA) responses were observed in these trials, which cause a reduced circulating half-life, thereby limiting their further use [30]. Therefore, SAgs targeting molecules with low antigenicity were suggested for the design of the next generation of SAgs drugs [13].

Tumour-related ligand is less antigenic than mAbs, and the conjugate composed of ligand and superantigen was presumed to kill one type of tumour [31]. However, native ligands are used less in targeting SAgs to tumours. This is largely due to internalisation induced by the ligand/receptor interaction, which prevents SAgs from being presented to the surface of tumour cells and activate T cells. To avoid the internalisation triggered by the binding of ligand to receptor, we use a mitogenic defective TGFα3L, instead of full length TGFα, as a targeting molecule for SEA_D227A_. Nevertheless, the affinity of TGFα3L with EGFR is weaker than that of full length TGFα, which raised a concern for its ability to bring superantigen to the tumour *in vivo*. As shown in this paper, TGFαL3SEA_D227A _can inhibit tumour growth in a EGFR-dependent way, implying that the affinity of TGFαL3 is sufficient for targeting the superantigen to the tumour. Meanwhile, we also noticed a decreased therapeutic effect of the fusion protein when a higher dose of fusion protein (600 pmol) was applied. This indicates that high dose treatment may result in increased nonspecific binding which eventually counteracts the tumour inhibitory effect of the fusion protein. The affinity of TGFαL3 to tumour should be further improved for better targeting efficiency. Despite that, the therapeutic effect of TGFαL3SEA_D227A _is still encouraging, especially when the dose was limited to concentrations between 6 pmol and 60 pmol.

**Figure 6:**
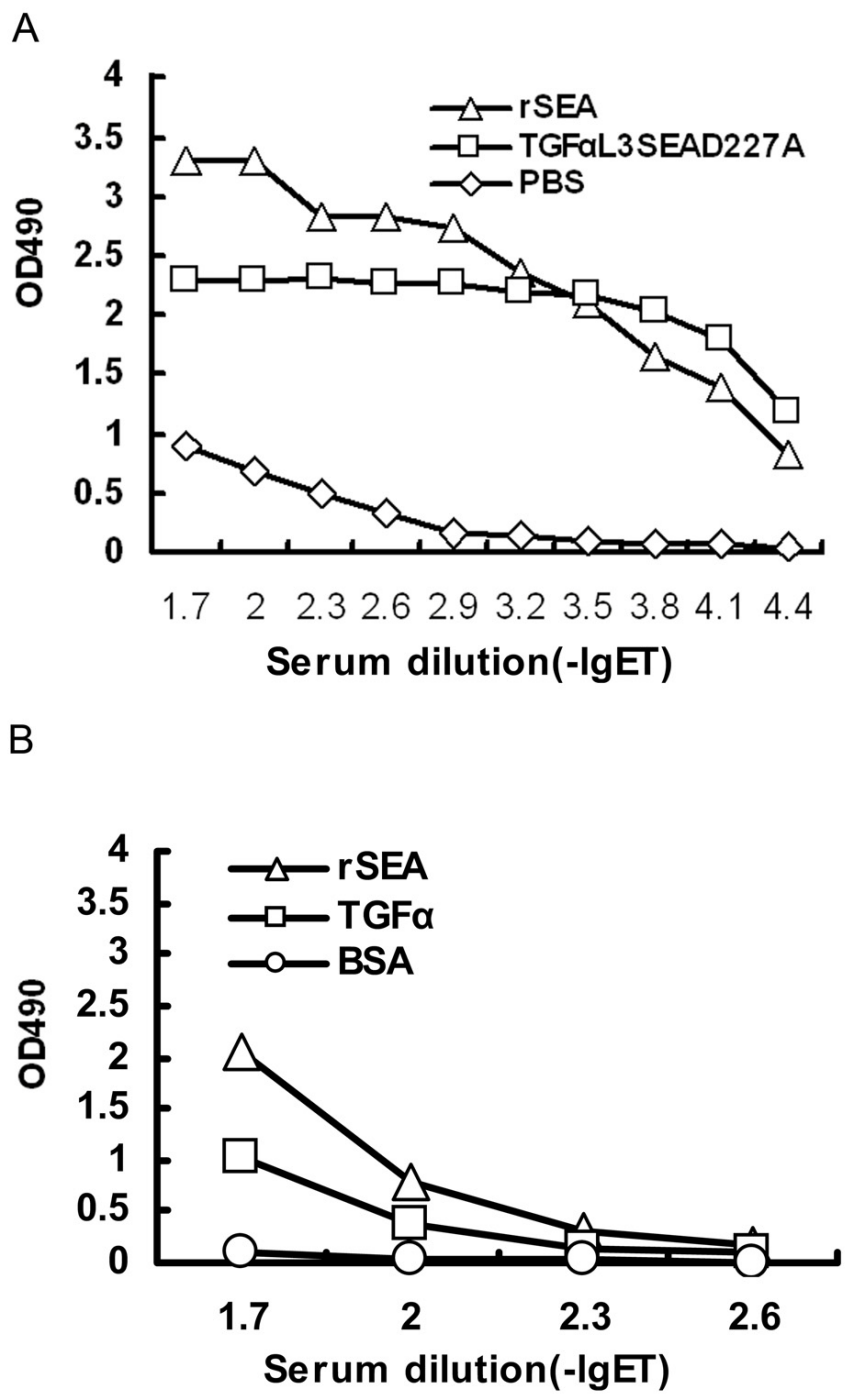
**The antigenicity evaluation of TGFαL3SEA_D227A_**. Nine mice were randomised into three groups and injected intramuscularly with 1 mg TGFαL3SEA_D227A_, rSEA, or PBS, respectively. The injections were repeated three times at two-week intervals. Then serum from each group was collected and analysed as follows. (A) To detect the antigenicity of TGFαL3SEA_D227A _(square) or rSEA (triangle), the corresponding proteins were coated on ELISA plates. The titres of antibody were detected in the standard way. (B) To analyse the antigen epitopes recognised by the TGFαL3SEA_D227A _antibody, the native ligand TGFα (square), rSEA (triangle), or BSA(circle)was coated and the anti-serum of TGFαL3SEA_D227A _was analysed in the same way as (A).

The antigenicity of TGFαL3SEA_D227A _is presumably lower than its monoclonal antibody orthologs. To test this hypothesis, the surviving mice in the TGFαL3SEA_D227A _treated group (at day 24 in the survival ratio test) were bled before being sacrificed and ELISA was used to detect antibodies against proteins of interest. No detectable titre was found in either high and low doses of TGFαL3SEA_D227A_-treated groups (data not shown). This result showed that the injection procedure for fusion protein in this paper could not induce apparent antibodies against these two proteins. However, we are not sure whether the same result will occur in the case of injection with a higher dose of fusion protein. It was reported that SAgs, including SEA and SEB, could act as an adjuvant to enhance humoral immunity via activating antigen specific CD4^+ ^T cells [32]. This observation raises the possibility that a higher dose of TGFαL3SEA_D227A _may boot the antibody against itself, owing to the adjuvant effect of SEA. To this end, we increased the injection dose to 1 mg and extended the interval between injections to two weeks. After three intramuscular injections, the titres of antibody against the proteins of interest were determined. As shown in Figure 6A, considerable titres of antibodies against both TGFαL3SEA_D227A _and rSEA were observed. Furthermore, the antibody against TGFαL3SEA_D227A _can also recognise TGFαL3 (Figure 6B). These observations confirmed the adjuvant property of SEA, indicating the inevitable antigenicity of superantigen macromolecules. Therefore, to reduce the antigenicity of SAgs macromolecules, the optimisation of both drug dose and treatment procedure will be very important when a preclinical trial is designed. Recently, it was reported that combinational use of Fab-SAgs fusion proteins with docetaxel not only improved the antitumour efficiency of Fab-SAgs but also reduced the production of anti-SEA antibody [33,34]. This finding provides another strategy to eliminate the antigenicity of SAgs macromolecules.

## Conclusions

The results in this paper showed that human TGFα-derived TGFαL3 is capable of directing SAgs to tumours and exerting an inhibitory effect on tumour growth, which makes TGFαL3SEA_D227A _an attractive candidate for immunotherapy on EGFR-expressing tumours.

## Methods

### Bacterial strains and Cell culture

*Escherichia coli *strain DH5α was used for plasmid propagation and cloning. Strain BL21 (DE3) (Novagen, Madison, WI, USA) was used as a host for the production of fusion proteins. A431 (epidermoid carcinoma) and B16 (a murine melanoma cell line) cells were maintained in Dulbecco's Modified Eagle medium (DMEM) (Gibco BRL, Life Technology, Rockville, MD) supplemented with 10% foetal bovine serum (FBS) (Gibco BRL, Life Technology, Rockville, MD), 2 mM L-glutamine, 100 units/ml penicillin and 100 mg/ml streptomycin. Mouse spleen cells, freshly separated from healthy C57BL/6 mice, were grown in DMEM/F12 cell culture medium.

### Plasmid Construction

A site mutation (D227A) in the SEA gene was introduced by PCR-amplification. This mutation resulted in the defective binding of SEA to the MHC-II molecule [23]. To avoid potential conformational perturbation caused by the fusion, an eight-amino acid flexible linker was also introduced between SEA_D227A _and TGFαL3. The primers for the PCR reaction include Primer 1, 5'-AA GGA TCC GGT GGT GGT AGC GAG AAA AGC-3', and Primer 2, 5'-ATG AAT TCA CTC GAG ACT TGT ATA TAA ATA TAT AGC AAT ATG CAT-3'. The PCR product was purified and digested with BamH I and Xho I. The digested SEA_D227A _gene was inserted into the pET-22b (+) vector (Novagen, Madison, WI, USA) to produce the plasmid pET-SEA_D227A_. To fuse TGFαL3 to the N terminal of SEA_D227A_, Primers 3 and 4 were annealed and extended (Primer 3: 5'-AA CAT ATG GTA TGC CAC TCT GGT TAC GTT GGC GCA CGT TGT GAA CAC-3'; Primer 4: 5'-TT GGA TCC AGA ACC ACC GAG CAG GTC AGC GTG TTC ACA ACG TGC GCC -3'). The resultant products were then digested by Nde I and BamH I and ligated into pET-22b-SEA_D227A _cut with the same restriction enzymes. The resultant recombinant vector was termed pET-22b-TGFαL3SEA_D227A_, and encodes TGFαL3 and SEA_D227A, _plus an eight-amino acid linker (GGSGSGGG) between them (Figure 1A).

To evaluate the influence of the TGFαL3 fusion direction on protein activity, another plasmid pET-22b-SEA_D227A_TGFαL3 was also generated, in which the TGFαL3 coding sequence was induced to the 3'-terminal of the SEA gene. First, a Sac I site was introduced into the 3'-terminal of the SEA gene by PCR using Primer 1 and Primer 5 (Primer 5: 5'-AA GTG GTG GAG CTC GAC ACT TGT A-3') and the PCR product was cloned into pET-22b, resulting in plasmid pET-22b-SEA_D227A_SacI. Then the cDNA encoding TGαL3 plus the eight-linker was generated by annealing and extending Primers 6 and 7 (Primer 6: 5'-AA GAG CTC GGT GGT GGT TCT GGT GGT GGT TCT GTA TGC CAC TCT GGT TAC GTT GGC-3'; Primer 7: 5'-AA CTC GAG GAG CAG GTC AGC GTG TTC GCA ACG TGC GCC AAC GTA ACC AGA GTG GCA -3'). The extension product and pET-22b-SEA_D227A_SacI were doubly digested by Sac I and Xho I and then ligated together. The resultant vector was named pET-22b-SEA_D227A_TGFαL3 (Figure 1A). All DNA constructs were confirmed by DNA sequencing. Both constructs were expected to express fusion proteins with 266 amino acids (aa), including TGFαL3 (17 aa), mutated SEA (233 aa), linker sequence (8 aa), C-terminal histidine tag (6 aa), and two amino acids (LE) before the histidine tag encoded by the restriction enzyme recognising sequence for cloning.

### Expression and purification of fusion proteins

Construct pET-22b-TGFαL3SEA_D227A _or pET-22b-SEA_D227A_TGFαL3 was transformed into BL21 (DE3) and then induced with 0.5 mM IPTG at 22°C overnight. The cell pellet was washed and suspended in binding buffer (0.5 mol/l NaCl, 0.1 mol/l Tris.Cl, pH8.0), followed by a regular sonication procedure. The cell debris was removed by centrifugation at 12,000 g for 30 min. The supernatant was then applied to Ni-charged chelating-Sepharose (Pharmacia Biotech, Uppsala, Sweden). After being washed with binding buffer plus 80 mM imidazole, the fusion protein was eluted with the binding buffer containing 250 mM imidazole and then dialysed against PBS buffer overnight. Fusion proteins were enriched by ultrafiltration and quantified by BCA assay (Pierce, Rockford, USA).

### Splenocyte proliferation assay

Splenocytes freshly isolated from the spleen of healthy C57BL/6 mice were seeded into 96-well plates at a density of 1 × 10^5 ^cells per well in the presence of 1.0 μg/ml TGFαL3SEA_D227A_, 1.0 μg/ml SEA_D227A_TGFαL3, 1.0 μg/ml rSEA, 25 μg/ml phytohemagglutinin(PHA) or 1.0 μg/ml BSA. The treated cells were cultured at 37°C for 72 h, then 20 μl 5 mg/ml 3-(4, 5-dimethylthiazol-2-μl)-2, 5- diphenyltetrazolium bromide (MTT) was added for another 4 h. Finally, 150 μl dimethyl sulphoxide (DMSO) was dispensed into each well and the optical density at 570 nm was measured on an ELISA reader (Biorad 550). The relative proliferating activity of the fusion protein was given by % 100 × OD570 of the fusion protein/OD570 of the rSEA.

### Cell ELISA assay

Binding of TGFαL3SEA_D227A _or SEA_D227A_TGFαL3 to EGFR was detected by cell ELISA [35]. Briefly, 1 × 10^4 ^A431 cells per well were partitioned into 96-well flat-bottomed plates overnight, then fixed with 10% neutral formaldehyde (10 mmol/l PBS, 10% formaldehyde, pH7.4) at room temperature for 1 h. The cells were blocked with 5 mg/mL bovine serum albumin (BSA) for 2 h and incubated with different concentrations of TGFαL3SEA_D227A_, SEA_D227A_TGFαL3 or rSEA at 37°C for 1 h. After being washed five times with PBST (10 mmol/l PBS pH7.4, 0.05%Tween-20), the cells were incubated with anti-hexahistidine MAb (1:1250), followed by HRP-conjugated rabbit anti-mice IgG (1:5000) and washed as previously. Finally, the colour was developed by 0.4 mg/ml orthopenylenediamine (OPD) solution with 1.5% H_2_O_2_, and the absorbance at 490 nm was analysed on ELISA reader.

### *In vitro *Tumour cell growth inhibition assay

Tests were performed according to the previously described method [36]. B16 cells or A431 cells in DMEM medium were added at a density of 2.5 × 10^4 ^cells/well to 96-well flat-bottomed plates, followed by treatment with the indicated reagents and effector cells to a total volume of 100 μl. Cells were cultured for 72 h at 37°C in 5% CO2. The remaining viable tumour cells were determined using the MTT assay. The data were given as the percentage of tumour cell growth inhibition (%TCGI), which was calculated as the following: %TCGI = 100 × (A_test_-A_b_)/(A_c_-A_b_). A_test _is the absorbance of tumour cells grown in the presence of the effector cells and various reagents, A_c _is the absorbance of the tumour cells grown in the medium, and A_b _is the absorbance of the medium only.

### *In vivo *tumour inhibition assay

C57BL/6 mice (4-6 weeks old) were obtained and maintained at the certified animal facility of Beijing Institute of Biotechnology. Fifty mice were inoculated subcutaneously with 1.0 × 10^6 ^B16 cells suspended in PBS plus 1% C57BL/6 mouse serum. When the tumour length exceeded 0.5 cm, the mice were randomly divided into five groups (10 mice per group) and subsequently injected intraperitoneally with 6 pmol (L), 60 pmol (M), 600 pmol (H) TGFαL3SEA_D227A_, 60 pmol rSEA or PBS four times at one day intervals. Tumour size was measured with calipers at the time points indicated. The tumour volume was calculated as (length × width^2^)/2 [37]. The tumour growth inhibition ratio was calculated as % 100 × (tumour volume of PBS control-tumour volume of interest)/tumour volume of PBS. The survival percentages of mice were analysed by Kaplan Meier Plot. All animal experiments were designed and conducted according to the recommendations of the Beijing Experimental Animal Regulation Board (SYXK/JING/2005/0031).

### Western blotting and Immunohistochemical analysis

Western blot analysis was performed routinely with the EGFR monoclonal antibody (Santa Cruz Biotechnology, Inc) or anti-ß actin polyclonal antibody (Santa Cruz Biotechnology, Inc). For immunohistochemical analysis, the tumour tissues from sacrificed mice were embedded in paraffin after being fixed with 10% formaldehyde in PBS (pH7.4). The sections were stained with mouse anti-CD4 (1:500) or anti-CD8 mAbs (1:400) (Millipore, Upstate, MA, USA) at 4°C overnight, followed by biotinylated secondary antibody (1:5000) for 10 min at room temperature. Finally, the colour was developed in a standard way.

## Authors' contributions

QX designed and performed experiments, analysis and manuscript writing. XZ conducted partial experiments and drafted the manuscript. JY collaborated on the design of the protein linker. CC provided partial reagents and suggestions in preparation for this manuscript. CL, QM, and HZ supervised the whole process. All authors have read and approved the final manuscript.
